# Strain-specific behavior of *Mycobacterium tuberculosis* in A549 lung cancer cell line

**DOI:** 10.1186/s12859-021-04100-z

**Published:** 2021-03-25

**Authors:** Shima Hadifar, Shayan Mostafaei, Ava Behrouzi, Abolfazl Fateh, Parisa Riahi, Seyed Davar Siadat, Farzam Vaziri

**Affiliations:** 1 Department of Mycobacteriology and Pulmonary Research, Pasteur Institute of Iran, Tehran, Iran; 2 Microbiology Research Center (MRC), Pasteur Institute of Iran, Tehran, Iran; 3 Department of Biostatistics, School of Health, Kermanshah University of Medical Sciences, Kermanshah, Iran; 4 Epidemiology and Biostatistics Unit, Rheumatology Research Center, Tehran University of Medical Sciences, Tehran, Iran; 5 Department of Biostatistics, Faculty of Medical Sciences, Tarbiat Modares University, Tehran, Iran

**Keywords:** *Mycobacterium tuberculosis*, Lung Cancer, Lineage, Elastic net penalized logistic regression

## Abstract

**Background:**

A growing body of evidence has shown the association between tuberculosis (TB) infection and lung cancer. However, the possible effect of strain‐specific behavior of *Mycobacterium tuberculosis* (*M.tb*) population, the etiological agent of TB infection in this association has been neglected. In this context, this study was conducted to investigate this association with consideration of the genetic background of strains in the *M.tb* population.

**Results:**

We employed the elastic net penalized logistic regression model, as a statistical-learning algorithm for gene selection, to evaluate this association in 129 genes involved in TLRs and NF-κB signaling pathways in response to two different *M.tb* sub-lineage strains (L3-CAS1and L 4.5). Of the 129 genes, 21 were found to be associated with the two studied *M.tb* sub-lineages. In addition, *MAPK8IP3* gene was identified as a novel gene, which has not been reported in previous lung cancer studies and may have the potential to be recognized as a novel biomarker in lung cancer investigation.

**Conclusions:**

This preliminary study provides new insights into the mechanistic association between TB infection and lung cancer. Further mechanistic investigations of this association with a large number of *M.tb* strains, encompassing the other main *M.tb* lineages and using the whole transcriptome of the host cell are inevitable.

## Background

Lung cancer and tuberculosis (TB) are the leading causes of human death and represent a global health concern. According to estimates from the 2018 Global Cancer Observatory (GLOBOCAN), lung cancer is the most frequent cancer, accounting for 11.6% of the 18.1 million newly diagnosed cancer cases [1]. In 2018, the World Health Organization (WHO) reported 10 million new cases of TB and 1.4 million TB-related deaths [2]. Different factors have been identified that might increase the risk of lung cancer [3]. Many risk factors are shared between lung cancer and TB infection [4]. Recent population-based studies have reported that the risk of lung cancer can increase with infection by *Mycobacterium tuberculosis* (*M.tb*), the etiological agent of TB infection [5–10]. An inflammatory microenvironment driven by cytokines, chemokines, and inflammatory cells during TB infection has been recognized as a process that can induce genetic and host tissue damage and contribute to carcinogenesis in lung tissue [11].

There is increasing evidence of a significant genetic diversity in the *M.tb* population [12, 13]. *M.tb* strain-specific host–pathogen interactions have been demonstrated in previous studies [14–18]. This characteristic may affect the trend of *M.tb* pathogenesis and the molecular mechanism behind the association between the risk of lung cancer and TB infection. However, the association between TB infection and lung cancer has been evaluated previously without considering the bacterial genotype. In the current study, we tried to provide new insight into this association with consideration of the genetic background of strains in *M.tb* population.

In Iran, the presence of remarkable diversity in *M.tb* population structure with predominance of L3-CAS1 and L4.5 (NEW1) sub-lineages has been documented. Epidemiologically, Iran has been identified as the probable origin of L4.5 and the ecological adaption and national occurring of this subpopulation was not unexpected. L3-CAS1sub-lineage is almost found in around the Indian Ocean and the influx of Afghan refugees may contribute to ongoing circulation of L3-CAS1sub-lineage in Iran. However, it seems genetic variability be the main driver of this epidemiological trend and transmission potential in both sub-lineages [13, 19, 20]. In line with, in our previous study, we compared these dominant sub-lineages of *M.tb* strains in interrupting TLRs and NF-κB signaling pathways in alveolar epithelial cell type II (A549 cell line) and observed strain-specific characteristics in interactions with host cells [16]. In the light of these results, we examined the gene expression profile of cancerous cell line in response to two *M.tb* with divergent genetic background by employing penalized statistical model and systems biology methods.

## Results

### Differential analysis of gene expression data

Based on the statistical comparisons by Limma R package, 37 genes were identified with significant down-regulation (all fold-regulations were less than − 2) compared to the *RPLP0* expression as the housekeeping gene, including *BTK, AKT1, CD86, CD80, IFNG, BCL3, TLR10, MAPK8IP3*, etc. In contrast, 25 genes were identified with a significant up-regulation (all fold-regulations were more than 2) compared to *RPLP0*, including *PTGS2(COX-2*)*, FOS, HSPA1A, NFKBIL1, MAP4K4, HSPD1, HSPA1A, FADD,* etc. (Table 1).

**Table 1:** The list of the differentially expressed genes in comparison with the housekeeping gene

Significantly down-regulated genes (n = 37)	Significantly up-regulated genes (n = 25)
*AKT1, BCL2L1, BCL3, BTK, CARD11, CD180, CD80, CD86, ELK1, HMGB1, PSIP1, PPARA, SIGIRR, TA**B1**,TBK1, TIRAP, TLR10, IFNG, IKBKG, IL1R1, IL2, IL10, IRF1, LTA, MAPK8IP3, TLR2, TLR4, TLR6, TLR7, TLR8, TNFRSF10A, TNFRSF1A, TRADD, TRAF2, TOLLIP, TRAF3, TNFAIP2*	*BCL2A1, CCL2, CCL5, CXCL2, CXCL8, EIF2AK2, FADD, FOS, PELI1, PTGS2, RELA, SARM1, HRAS, HSPA1A, HSPD1, HMOX1, IRF3, JUN, MAP2K4, MAP3K1, MAP4K4, NFKBIA, NFKBIL1, NFRKB, NR2C2*

### Gene selection and literature search

**Fig. 1:**
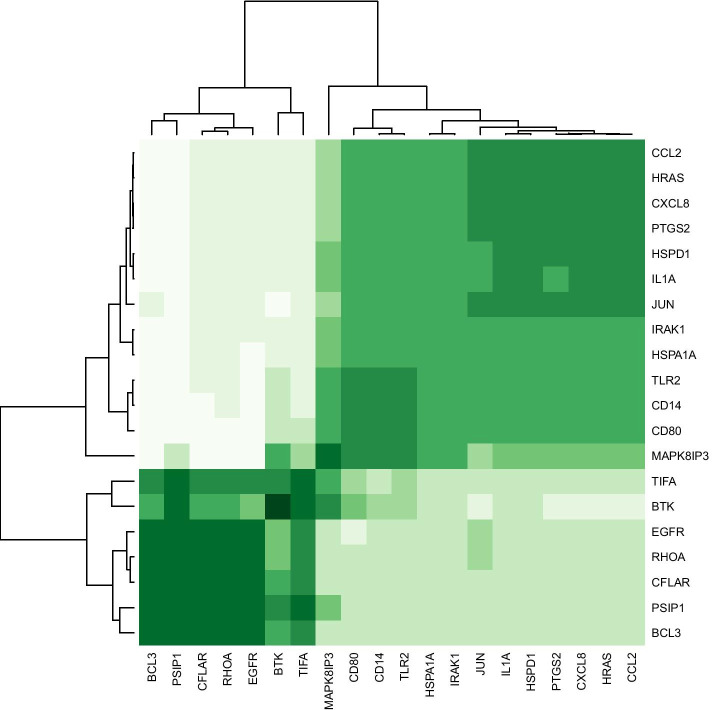
Spearman's rank correlation matrix for co-expression between the selected genes: heatmap for agglomerative hierarchical clustering of the 21 selected genes based on their patterns of gene expression. The *IFNG* gene is not correlated with other genes and has not shown in the heatmap

**Fig. 2:**
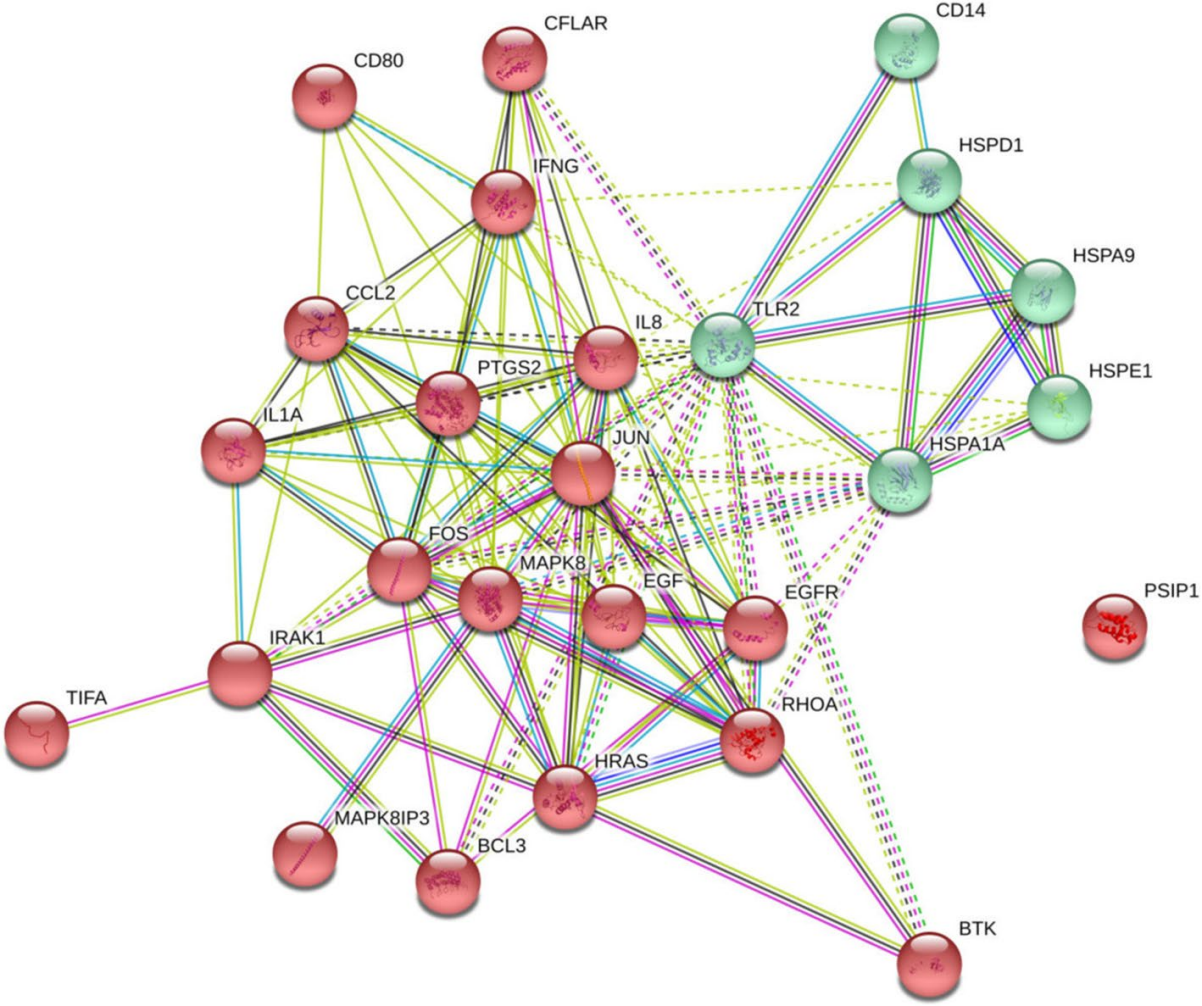
STRING protein–protein interaction networks for the 21 selected genes

By using elastic net regularized logistic regression as a statistical-learning algorithm for gene selection, 21 genes were selected all in association with either of the two *M.tb* sub-lineages (Table 2). The most important selected genes included *CFLAR, TIFA, HRAS, IRAK1, CD14, BTK, MAPK8IP3, IFNG, JUN, IL1A, CD80, PSIP1, PTGS2, BCL3, HSPA1A, RHOA, CCL2(MCP-1), CXCL8, HSPD1, TLR2*, and *EGFR*. The importance values of all the selected genes were more than 20%. Co-expression hierarchical heatmap between the selected genes was presented using Spearman's rank correlation matrix (Fig. 1). As shown in Fig. 1, the selected genes were clustered in the two main clusters based on the co-expression pattern. According to the co-expression patterns of the selected genes, *TIFA, BTK, EGFR, RHOA, CFLAR, PSIP1,* and *BCL3* had a similar expression pattern while the other selected genes formed another group with the same expression patterns (e.g. *CCL2, HRAS, CXCL8, PTGS2, HSPD1, IL1A,* and *JUN*). Figure 2 displays the functional protein-association networks for the 21 selected genes.

**Table 2:** Genes selected by elastic-net regularized multinomial logistic regression for the association between the genes and the dominant genotypes of *M.tb* strains

	Gene Symbol	Elastic net	Fold Regulation (L3-CAS1 vs. Con.)	Adjusted P-value (L3-CAS1 vs. Con.)	Fold Regulation (L4.5 vs. Con.)	Adjusted *p* value (L4.5 vs. Con.)	Fold Regulation (L4.5 vs. L3-CAS1)	Adjusted *p* value (L4.5 vs. L3-CAS1)
1	*CFLAR*	100%	− 1.544	0.082	1.376	0.117	2.125	0.044
2	*TIFA*	93.9%	− 1.392	0.199	− 1.206	0.399	1.154	0.256
3	*HRAS*	91.8%	3.279	0.0001	6.681	0.0001	2.038	0.001
4	*IRAK1*	82.36%	1.395	0.082	1.136	0.503	− 1.228	0.256
5	*CD14*	77.93%	4.724	0.0001	− 1.217	0.171	− 5.749	0.0001
6	*BTK*	67.28%	− 4.258	0.055	− 440.602	0.001	− 103.476	0.0001
7	*MAPK8IP3*	59.63%	− 13.117	0.0002	− 12.323	0.0003	1.064	0.114
8	*IFNG*	55.51%	− 24.028	0.0001	− 9.426	0.0002	2.549	0.001
9	*JUN*	41.91%	3.146	0.0001	12.582	0.0002	3.999	0.001
10	*IL1A*	39.77%	2.204	0.0001	3.160	0.0001	1.434	0.081
11	*CD80*	33.64%	− 3.630	0.0001	− 5.856	0.0002	− 1.613	0.044
12	*PSIP1*	31.03%	− 3.972	0.045	− 2.075	0.126	1.914	0.011
13	*PTGS2*	29.73%	40.880	0.0001	84.449	0.0001	2.066	0.001
14	*BCL3*	26.79%	− 12.098	0.035	− 1.617	0.188	7.482	0.0001
15	*HSPA1A*	26.63%	10.581	0.0001	4.009	0.0002	− 2.639	0.001
16	*RHOA*	26.27%	− 1.245	0.055	1.526	0.003	1.900	0.011
17	*CCL2*	26.23%	2.023	0.045	5.993	0.0002	2.962	0.001
18	*CXCL8*	24.92%	6.364	0.0001	20.966	0.0001	3.294	0.0001
19	*HSPD1*	23.79%	7.345	0.0001	9.759	0.0002	1.329	0.114
20	*TLR2*	22.52%	− 8.980	0.0001	1.651	0.0017	14.826	0.0001
21	*EGFR*	21.83%	− 3.547	0.035	1.286	0.433	4.561	0.0001
Misclassification error rate		14.8%	–	–	–	–	–	–
lambda		0.00849	–	–	–	–	–	–
Alpha		0.50	–	–	–	–	–	–

Lambda is the tuning parameter and it is for controlling the overall strength of the penalty. Alpha is a controlled parameter between LASSO (L1) and ridge (L2) penalties. Alpha = 0.5 corresponds to elastic-net regularization. Importance value for each selected gene was reported, *p* values adjusted by Benjamini-Hochberg-FDR correction at α = 0.05

Furthermore, results of the literature search demonstrated 20 out of the 21 genes had been previously reported in the literature to be associated with lung cancer or lung function diseases (Table 3). We identified *MAPK8IP3* as a novel gene, which has not been previously detected in lung function studies. These selected genes may deserve special attention for the future development of therapeutics intervention.

**Table 3:** Confirmation of the associations of 21 selected genes with lung cancer/or lung function by literature reviewing in PubMed with keywords ((“Lung Cancer” OR “Lung Function”) AND “name of each selected gene”) in the title and abstract fields

Gene Symbol	Number of studies	References (PMIDs)
*CFLAR*	8	29,258,089, 29,165,042, 27,186,395, 24,387,758, 21,430,781, 20,713,531, 19,860,666, 17,450,141
*TIFA*	1	29,975,933
*HRAS*	37	30,196,239, 29,641,535, 29,636,358, and etc
*IRAK1*	4	25,550,857, 29,667,303, 26,262,504, 24,144,839
*CD14*	100	30,325,558, 30,112,108, 29,343,695, 29,448,000, and etc
*BTK*	5	28,990,652, 28,734,581, 27,437,104, 27,111,445, 17,327,079
*MAPK8IP3*	0	-
*IFNG*	12	29,516,506, 24,244,422, and etc
*JUN*	200	29,262,633, 29,858,032, and etc
*IL1A*	3	25,554,695, 12,752,325, 12,737,276
*CD80*	30	11,422,893, 26,702,740, and etc
*PSIP1*	1	24,670,920
*PTGS2*	22	22,761,909, 22,464,751, and etc
*BCL3*	2	20,420,878, 26,122,346
*HSPA1A*	5	22,037,874, 29,137,250, 28,331,811, 26,093,302, 20,404,511
*RHOA*	100	29,995,590, 29,337,059, 28,888,686, and etc
*CCL2*	66	30,061,946, 29,892,522, 29,722,145, and etc
*CXCL8*	55	27,578,214, 29,636,079, 28,836,545, and etc
*HSPD1*	5	28,978,099, 28,698,656, and etc
*TLR2*	32	27,097,965, 22,952,638, 24,630,931, and etc
*EGFR*	3692	30,350,450, 30,349,313, 30,343,004, and etc

### Comparison of selected genes expression between *M.tb* L4.5 and L3-CAS1 sub-lineages groups

We investigated the differential expression of the 21 selected genes between *M.tb* L4.5 and L3-CAS1 sub-lineages groups. A comparison of *M.tb* L4.5 to L3-CAS1 sub-lineages showed significant differences in the expression of 13/21 (61.9%) genes. Among these 13 genes, *CFLAR, HRAS, IFNG, JUN, PTGS2, BCL3*, *CCL2, CXCL8, TLR2,* and *EGFR* were significantly up-regulated in the L4.5 sub-lineage group compared to the L3-CAS1 sub-lineage group, while *CD14, BTK*, and *HSPA1A* genes were significantly down-regulated in the *M.tb* L4.5 sub-lineage group compared to the *M.tb* L3-CAS1 sub-lineage group (Table 2). In addition, *MAPK8IP3* was not changed in the *M.tb* L4.5 sub-lineage group compared to the *M.tb* L3-CAS1 sub-lineage group (adj. P = 0.114).

## Discussion

In the present study, we investigated lung cancer-related genes that differentially were regulated by different genotypes of *M.tb* in lung adenocarcinoma cell line using the statistical penalized algorithm. In our analyses, we identified 21 potentially lung cancer-related genes during infection with *M.tb* L3-CAS1 and L4.5 sub-lineages.

Various inflammatory processes and functional pathway-associated genes, which are involved in carcinogenesis, have been investigated in different studies. Chemokines secretion is one of the main ways for recruitment of host cell and inhibition of antitumor immune responses in cancerous cells [21, 22]. MCP-1 is one of the chemotactic stimuli that is secreted from cancerous cells and induces immunosuppressive microenvironments [23]. There are some controversies about the role of this chemokine in lung cancer pathogenesis [24, 25]. However, Fridlender et al. found that the blockade of *MCP-1* could inhibit lung tumorigenesis and could be proposed as a promising approach to lung cancer treatment [26]. Besides, the role of IL8 as another chemokine, which typically plays a role in the induction of angiogenesis and its overexpression, has been reported in lung cancer. In addition, the overexpression of *COX-2*, an inflammation-associated gene, has been found in different stages of lung cancer [27]. This up-regulation may be explained the functional role of this gene in lung tumorigenesis and prediction of patient outcomes. In line with lung cancer studies, in our analysis, the expression of *MCP-1, IL-8,* and *COX-2* was up-regulated in the cancerous cell line in response to the infection with *M.tb* L4.5 sub-lineage when compared to the infection with *M.tb* L3-CAS1 sub-lineage (*p* < 0.001).

IFN-γ is generally considered as a cytokine with antitumor activity. However, there are significant controversies about the role of this cytokine. Increasing evidence suggested that IFN-γ may have dual aspects in its function and act as both an anti-tumorigenic and a pro-tumorigenic cytokine [28]. The pro-tumorigenic property of IFN-γ is based on the upregulation of immunosuppressive cells such as Treg cells and Th17 [29]. The pivotal role of this cytokine in regulating the Programmed death-ligand 1 (*PD-L1*) gene expression, as a factor that has an inhibitory role in cancer immunity, and promoting the immune evade has been found in tumor cells [30]. Besides, the upregulation of PD-L1 expression and induction of lung carcinoma by IFN-γ have been revealed [30]. Similarly, in vitro* and *in vivo concomitant H37Rv infection in non-small cell lung cancer showed that lung cancer progression facilitated by enhancing Treg cells proportion and the upregulation of PD-L1 expression that induced by H37Rv as a part of *M.tb* lineage 4 [31]. In the current study, based on the expression of IFN-γ in response to the infection with *M.tb* L4.5 sub-lineage, compared to L3-CAS1 sub-lineage, this expression profile might be favor for better control of *M.tb* L4.5 sub-lineage strain compared to L3-CAS1 sub-lineage in infected host cell, in addition may contribute to the pathogenesis and deterioration of lung cancer during infection with *M.tb* L4.5 sub-lineage strain. Evaluating the expression level of *IL-8*, *MCP-1* and *IFN-γ* genes in A549 cell line in response to infection with F15/LAM4/KZN(LAM sub-lineage), F11(LAM sub-lineage), F28 (S sub-lineage) and Beijing (L2-Beijing sub-lineage) genotypes showed that the higher level of upregulation in the both genes are induced in response to infection with *M.tb* LAM sub-lineages compared to the other sub-lineages[32],While they failed to detect the expression of *IFN-γ* in response to all strains. However, the *M.tb* L4.5, LAM and S strains are members of lineage 4, differentially induced host response. The strain‐specific characteristic of *M.tb* population may have the potential to be considering in lung cancer cells studies.

Deregulation of apoptosis and cell proliferation pathways are the key mechanisms playing important roles in cancer pathogenesis [33]. The blockade of apoptosis can be mediated by the overexpression of anti-apoptotic proteins such as FLIP. The upregulation of FLIP also has been detected in lung carcinoma [34, 35]. In line with lung cancer study, the expression of *FLIP* was upregulated in response to L4.5 sub-lineage when compared with L3-CAS1 sub-lineage in our study (*p* < 0.05). The overexpression of FLIP by *M. tb* L4.5 sub-lineage may contribute to exacerbation of lung cancer during infection with this strain. This overexpression and inhibiting of the apoptosis is also favor for *M.tb* pathogenesis.

In addition, it has been demonstrated the blockade of the Rho/Rho-kinase pathway, which is involved in cancer proliferation and invasion, inhibited tumor migration and invasion [36, 37]. The knockdown of *RhoA* as the member of the Rho family inhibits lung cancer cell proliferation and induces apoptosis [38]. In our study, the expression of *RhoA* did not change in response to L4.5 sub-lineage when compared with L3-CAS1 sub-lineage. In addition, the deregulation of the *HRAS* gene, which is involved in the proliferation of different cancers, has been reported [39, 40]. Overexpression of the *Ras* oncogene family member was identified in response to L4.5 sub-lineage when compared with L3-CAS1 sub-lineage. This upregulation can contribute to the progression of cancerous cells.

Among the 21 selected genes, *BCL3* has an inhibitory function. The deregulation of this gene as an atypical member of the IκB family has been shown in different solid tumors[41]. In addition, Dimitrakopoulos et al*.* described the role of this gene in lung carcinogenesis [42]*.* They reported an increase in *BCL3* expression in lung cancer. This overexpression could be directly related to the increased level of *EGFR* expression. Aberrant *EGFR* expression are implicated in the progression of malignant cells manner [43, 44]. *EGFR* can promote angiogenesis by upregulation of main angiogenesis mediators such as Vascular endothelial growth factor (*VEGF*). Angiogenesis plays important role in the solid tumors growth and metastasis spreading [45]. Moreover, the expression of *HDM2* as a negative regulator of p53 that is a tumor suppressor gene was induced by the upregulation of *BCL3*[46]. In our analysis, based on the expression of *BCL3* and *EGFR* in response to L4.5 sub-lineage when compared to L3-CAS1 sub-lineage, we hypothesize that infection by the L4.5 sub-lineage strain may be potent to deteriorate lung carcinoma by promoting tumor growth and angiogenesis.

Besides, it has been proposed that *HSPA1A (HSP70*), a chaperone molecule, is strongly involved in promoting and development of different tumor cells and overexpression of this heat-shock protein has been shown to be associated with the progression of several tumors such as lung cancer [47]. The current findings were consistent with the previous studies. However, level of *HSP70* expression in response to infection with *M.tb* L4.5 sub-lineage was lower in compared to L3-CAS1 sub-lineage.

It is noteworthy that some inconsistent results were found in the current study. It has been shown TLRs pathway molecules such as *IRAK1*and *TLR2* have important roles in neoplasm diseases [48] and the significant upregulation of *IRAK1* and its involvement in the development of solid tumors including lung cancer have been reported [49, 50]. Besides, high expression of *TIFA* [51] and *IL1A* as a gene, which regulates tumor growth, angiogenesis, and metastasis in lung carcinoma cell has been reported. [52]. Contrary, no changes in the expression of all aforementioned genes were observed during infection with *M.tb* L4.5 sub-lineage compared to L3-CAS1 sub-lineage. Although the expression of the genes is controversial, the expression profile of other genes suggested the possibility that infection with the *M.tb* L4.5 sub-lineage strain drive cancer cell to progression. In the other word, the risk of progression might have promoted in lung cancer patients with lung that infected by *M.tb* L4.5 sub-lineage strain compared to *M.tb* L3-CAS1 sub-lineage strain. These patients also are more potent to secondary infections.

In contrast to our results, Mvubu et al. [32] showed that the expression level of *IRAK1* and *IL1A* were increased in response to infection with LAM sub-lineages(F15/LAM4/KZN, F11), S sub-lineage (F28) and Beijing sub-lineage. Level of this increase was higher in response to LAM sub-lineages compared to the other sub-lineages. It is possible that infection with LAM sub-lineages similar to L4.5 sub-lineages is more potent to drive cancer cell to progression.

In our analysis, we also identified *MAPK8IP3* as a novel and potent target that has not been reported in previous lung cancer studies. *MAPK8IP3* is a scaffold gene, also known as JIP3, that exhibits function in the JNK pathway[53]. The overexpression of this gene has been shown in different tumor cells [53, 54]. Therefore, *MAPK8IP3* may have the potential to be recognized as a novel biomarker in lung cancer investigation.

Based on the results of previous studies that demonstrated elastic net penalized logistic regression frequently performed better than Ridge, LASSO, and some statistical-based learning algorithms for model selection consistency and prediction accuracy [55], the use of this modern and accepted computational method in high dimensional gene expression data is a strength of the current study. The validation of all the results by literature review, the use of an appropriate cross-validation method (repeated 5-CV), the address of potential sources of bias and the use of STRING networks are the other strengths of the present study. However, the main limitations of our study are that the cell line selection was confined to adenocarcinoma of lung cell line and protein levels of selected genes were not assessed.

## Conclusions

The evidence of epidemiological association between TB infection and lung cancer is well established. This preliminary study provides new insights into the mechanistic association between TB infection and lung cancer. The two studied *M.tb* sub-lineages promoted cancer development by creating an inflammatory environment through differentially down/up-regulation of gene involved in TLRs and NF-κB signaling pathways. This environment has crucial impact on cell proliferation, apoptosis and angiogenesis. Based on significant strain‐specific behavior of *M.tb* population in host–pathogen interactions and according to our findings, investigation of linking TB infection to lung cancer in the context of the genetic background of *M.tb* strains might be more effective to gain a better understanding of this association, identification of *M.tb* strain‐specific behavior and therapeutic intervention. Further investigations with a large number of *M.tb* strains, encompassing the other main *M.tb* lineages and using the whole transcriptome of the host cell are inevitable. However, providing further information to fully understand of significant *M.tb* strain‐specific behavior related to lung cancer progression and minimizing bias are needed by means of high throughput methods.

## Methods

### Study design

The study was designed in accordance with our previous study [16] which investigated the gene expression profile of infected A549 cell line (ATCC CCL‐185) in response to dominant genotypes of *M.tb*. Briefly, the dominant genotypes of *M.tb* (L3-CAS1 and L4.5 strains) in the capital of Iran were identified based on 24 loci MIRU-VNTR and Spoligotyping[19] and confirmed by whole genome sequencing method. Then, the A549 cell line (maintained in antibiotic- free media) was infected in triplicates with the dominant genotypes an multiplicity of infection (MOI) of ~ 50:1 (50 bacteria:cell) for 72 h supplemented Dulbecco’s modified Eagle medium (DMEM) and After the time, cellular response involved in TLRs and NF-κB signaling pathways was evaluated by qRT‐PCR. RT^2^ Profiler™ PCR Array kits (QIAGEN), which include RT^2^ Profiler™ PCR Array Human Toll‐Like Receptor Signaling Pathway (QIAGEN, Cat.No. PAHS‐018ZF‐2) and RT^2^ Profiler™ PCR Array Human NF‐κB Signaling Pathway (QIAGEN, Cat.No. PAHS‐025YF‐2) according to the manufacturer’s instructions was used to perform qRT‐PCR. The expression of 168 pathway‐specific genes was evaluated and 39 genes were shared between these pathways. Secretion level of 12 cytokines/chemokines was assessed by ELISA arrays kit (QIAGEN). Viability of infected and mock cells was evaluated by the trypan blue exclusion test based on the manufacturer’s instructions (Sigma Aldrich, Germany). In addition, intracellular growth assay and intracellular internalization index were carried out [16].

### Gene expression analysis

The comparative cycle threshold (Ct) method (2^−ΔCt^ × 10^3^) was used to demonstrate the relative gene expression across the samples and the fold change was calculated using the 2^−ΔΔCt^ method [56]. Next, the primary gene expression data were qualified and normalized. Linear modeling for statistical comparison was applied by “limma” R package [57]. The cutoff of the false discovery rate for statistical comparison between the control and TB groups was considered at the level of 0.10.

### Gene selection model

Elastic net regularization produced a sparse model with good prediction accuracy and good grouping capability. Elastic net frequently has served better than the Ridge, LASSO, and many other statistical learning algorithms in gene selection consistency and prediction accuracy in gene datasets [55, 58]. Elastic net is introduced as a compromise between these two techniques, combining strength between the Ridge and LASSO penalized regression [59]. The elastic net penalized logistic regression was performed by “glmnet” R package (https://cran.r-project.org/web/packages/glmnet). The two *M.tb* sub-lineages were considered as dependent variable and expression level of the 129 genes were considered as independent/ or predictive variables in the elastic net regularized logistic regression for gene selection. The importance value of each selected gene was calculated using “varImp” function in “Caret” R package. Interactive agglomerative hierarchical clustering heatmap was applied by “heatmaply” R package in order to draw the co-expression heatmap between the selected genes (https://cran.r-project.org/web/packages/heatmaply). Statistical significance was considered at the level of 0.05 in the all of statistical methods.

### Cross-validation and literature validation

In order to validate the performance of the elastic net penalized regression, the repeated fivefold cross-validation was used. The model split the dataset by using repeated random sub-sampling with 100 repetitions in the fivefold cross-validation, permuting the sample labels every time. The cross-validated performance was summarized by observed misclassification error rate. In addition, to assess the literature validation for any result, a literature mining was used in PubMed by the search strategy of (“Lung Cancer” OR “Lung Function”) AND (“name of each selected gene”) and related MeSH terms in title and abstract fields.

## Data Availability

The datasets used and/or analyzed during the current study could become available through the corresponding author on reasonable request.

## Abbreviations

Ct: Cycle threshold
GLOBOCAN: Global Cancer Observatory
*M.tb*: *Mycobacterium tuberculosis*
*PD-L1*: Programmed Death-Ligand 1
TB: Tuberculosis
